# Recurrent Excitatory Feedback From Mossy Cells Enhances Sparsity and Pattern Separation in the Dentate Gyrus *via* Indirect Feedback Inhibition

**DOI:** 10.3389/fncom.2022.826278

**Published:** 2022-02-10

**Authors:** Alessandro R. Galloni, Aya Samadzelkava, Kiran Hiremath, Reuben Oumnov, Aaron D. Milstein

**Affiliations:** Department of Neuroscience and Cell Biology, Center for Advanced Biotechnology and Medicine, Robert Wood Johnson Medical School, Rutgers University, Piscataway, NJ, United States

**Keywords:** neuronal circuits, computational modeling, dentate gyrus, pattern separation, sparse coding, mossy cells, interneurons, cellular diversity

## Abstract

It is generally appreciated that storing memories of specific events in the mammalian brain, and associating features of the environment with behavioral outcomes requires fine-tuning of the strengths of connections between neurons through synaptic plasticity. It is less understood whether the organization of neuronal circuits comprised of multiple distinct neuronal cell types provides an architectural prior that facilitates learning and memory by generating unique patterns of neuronal activity in response to different stimuli in the environment, even before plasticity and learning occur. Here we simulated a neuronal network responding to sensory stimuli, and systematically determined the effects of specific neuronal cell types and connections on three key metrics of neuronal sensory representations: sparsity, selectivity, and discriminability. We found that when the total amount of input varied considerably across stimuli, standard feedforward and feedback inhibitory circuit motifs failed to discriminate all stimuli without sacrificing sparsity or selectivity. Interestingly, networks that included dedicated excitatory feedback interneurons based on the mossy cells of the hippocampal dentate gyrus exhibited improved pattern separation, a result that depended on the indirect recruitment of feedback inhibition. These results elucidate the roles of cellular diversity and neural circuit architecture on generating neuronal representations with properties advantageous for memory storage and recall.

## 1. Introduction

A prerequisite for highly similar experiences to be stored in the brain as distinct memories that can be independently recalled is for different combinations of sensory inputs to produce distinct patterns of neuronal activity. This important function of neuronal circuits is termed “pattern separation,” and it is thought that a brain region in mammals called the hippocampus subserves this function as part of a larger role in the storage and recall of spatial and episodic memories (Burgess et al., 2002; Leutgeb et al., 2007; Yassa and Stark, 2011). The input layer to the hippocampus is called the dentate gyrus, and it is characterized by extremely sparse and selective neuronal activity patterns during active spatial exploration in rodents. There are greater than 10-fold more primary output neurons in the dentate gyrus (the granule cells) than projection neurons in the entorhinal cortex that provide the major excitatory input to the dentate. Recent work has shown that during spatial exploration of a given environment, ~70% of cortical inputs are active, often at multiple locations within the environment, while only ~2–5% of dentate granule cells are active, typically at a single location (Jung and McNaughton, 1993; Senzai and Buzsáki, 2017; Hainmueller and Bartos, 2018; Cholvin et al., 2021). In this study we use computational modeling to investigate the neural circuit mechanisms that support this transformation from dense and overlapping combinatorial patterns of activity in cortex into ultrasparse, unique patterns of activity in the hippocampus.

In addition to primary excitatory output neurons, neuronal circuits in the hippocampus and cortex typically include numerous classes of local inhibitory interneurons (Tremblay et al., 2016; Pelkey et al., 2017). Inhibition from interneurons that receive the same incoming afferent inputs as the output neurons is termed “feedforward inhibition," and inhibition from cells that receive input from the output neurons themselves is called “feedback inhibition.” These classes of interneurons have been implicated in specific computational functions in neural circuits. One important function proposed for feedforward inhibition is “background subtraction,” whereby inhibition grows in proportion to and cancels the average level of input to the circuit, enabling only large fluctuations in inputs above the average level to drive circuit output (Grienberger et al., 2017; Rennó-Costa et al., 2019). Here we ask whether this background subtraction mechanism can support a constant level of output given a wide range in the total number of active inputs.

Feedback inhibition has been proposed to regulate the maximum number of output neurons that respond to the same pattern of input (de Almeida et al., 2009; Stefanelli et al., 2016; Rennó-Costa et al., 2019). This circuit function has been termed “winner-take-all,” or “lateral” inhibition, whereby the neurons receiving the highest level of excitatory input recruit feedback inhibition that suppresses neighboring neurons which are receiving less excitation. However, previous modeling work has shown that feedback inhibition alone is not able to prevent the number of active output neurons from increasing as the total amount of afferent input grows (Rennó-Costa et al., 2019). Furthermore, it is not clear if the extremely low fraction of active output neurons in circuits with ultrasparse representations like the hippocampal dentate gyrus is sufficient to activate the level of feedback inhibition necessary to support “winner-take-all” competition.

Another neuronal cell type that is present in the dentate gyrus may provide a solution to this conundrum—the mossy cell. These somewhat atypical neurons are excitatory interneurons—they form recurrent synapses within the dentate gyrus that contact inhibitory interneurons, the excitatory granule neurons, and other mossy cells, but do not send projections downstream to other circuit layers. Mossy cells receive their primary excitatory input from granule cells, categorizing them as “feedback excitatory” (FB Exc) neurons (Scharfman and Myers, 2013; Scharfman, 2016, 2018; Sun et al., 2017; Li et al., 2021; Ma et al., 2021). In contrast to dentate granule cells, mossy cell activity is less sparse and less selective, with most mossy cells active at multiple positions in space and in multiple distinct environments (Danielson et al., 2017; GoodSmith et al., 2017; Senzai and Buzsáki, 2017). This could be in part due to the recurrent connectivity between mossy cells which could serve to amplify and self-sustain a high degree of activity (Ma et al., 2021).

Since mossy cells both directly excite granule cells, and indirectly inhibit them by recruiting feedback inhibition (Scharfman, 1995), it has not been clear whether the net impact of mossy cells on granule cells is excitatory or inhibitory. However, recent work has shown that optogenetic activation of mossy cells dampens hippocampal excitability in epileptic mice (Bui et al., 2018), supporting an important role for mossy cells in recruiting inhibition. In this study, we tested the hypothesis that mossy cells provide the excitatory drive to feedback inhibitory cells necessary to support competition between granule cells under conditions when too few granule cells are active to recruit feedback inhibition on their own. We constructed a simple network model comprised of threshold linear rate neuronal units with conductance-based synapses, and simulated neuronal responses to a combinatorial set of stimuli with a wide range in the number of active inputs. To investigate the impact of specific cell types and connections on sparsity, selectivity, and discriminability of neuronal output activity patterns, we systematically compared network configurations comprised of different combinations of feedforward and feedback inhibitory and excitatory cells. We found that, even when synaptic connection strengths are initialized randomly without learning, incorporation of biologically realistic neuronal cellular diversity and connectivity enabled highly divergent activity patterns to be generated from largely overlapping patterns of input.

## 2. Results

### 2.1. Separating Patterns With a Wide Range of Input Activity Levels

**Figure 1:**
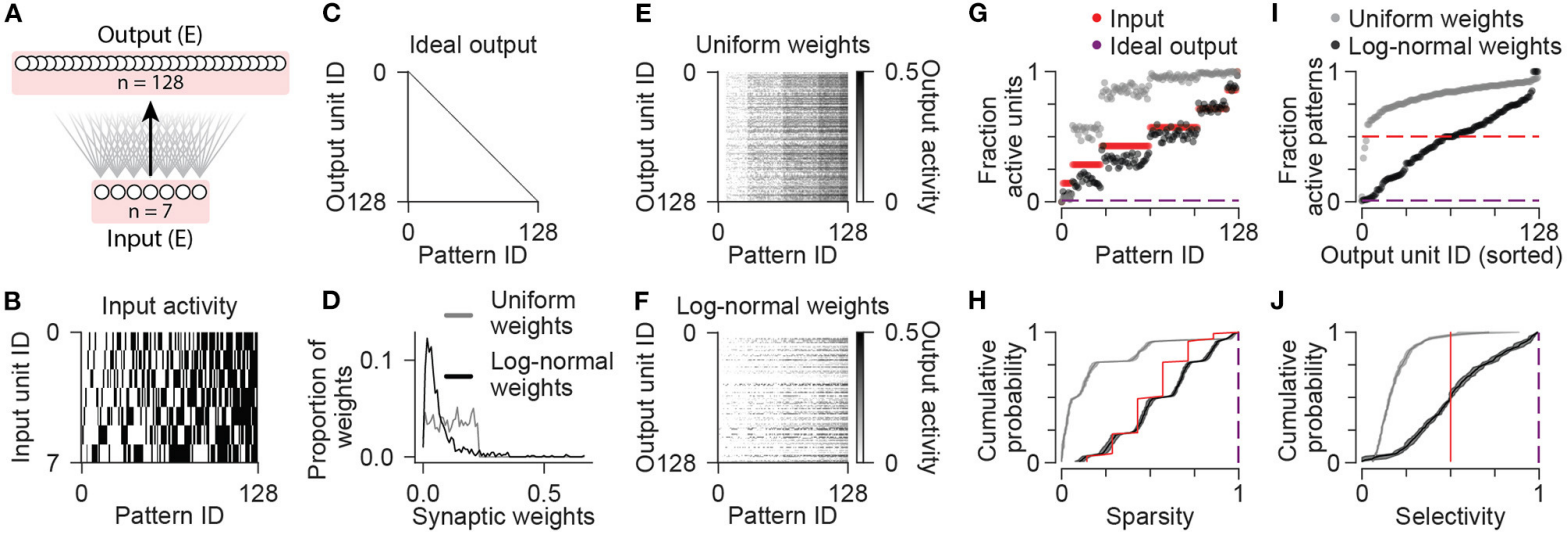
Sparsity and selectivity in a simple neuronal network model. **(A)** Diagram illustrating the connectivity of a simple model network containing only excitatory neurons. **(B)** Patterns of activity consisting of all possible combinations of 7 binary input units. **(C)** Idealized pattern of output where each input pattern is represented uniquely by a single output unit. **(D)** Synaptic weights from input to output units sampled from uniform (gray) and log-normal (black) distributions. **(E)** Activity of output units in the network with uniform synaptic weights in response to all input patterns. **(F)** Same as **(E)** for the network with log-normal synaptic weights. **(G)** For each input pattern, the fraction of the population with nonzero activity is shown for input units (red), and output units in the network with uniform (gray) or log-normal (black) synaptic weights. Ideal fraction active output (purple) is shown for reference. **(H)** Population sparsity across all input patterns is shown as cumulative probability distributions (Uniform vs. Input: *p* < 0.001; Log-normal vs. Input: *p* < 0.001; Log-normal vs. Uniform: *p* < 0.001). Ideal sparsity (purple) is shown for reference. **(I)** For each unit in a population, the fraction of patterns with nonzero activity is shown. Units are sorted by their responsiveness. For comparison, dashed lines indicate the fraction of active units for input units (red) and idealized output. Data shown in **(D–G,I)** are from single representative instances of the network. **(J)** Pattern selectivity across all units is shown as cumulative probability distributions (Uniform vs. Input *p* < 0.001; Log-normal vs. Input: *p* < 0.001; Log-normal vs. Uniform: *p* < 0.001). Ideal sparse output (purple) is shown for reference. In **(H,J)**, solid lines and shading indicate mean and standard deviation across five network instances. Statistical comparisons reflect two-sample two-tailed Kolmogorov-Smirnov tests with *p*-values adjusted by Bonferroni correction for multiple comparisons.

To explore the impact of specific neural circuit elements on pattern separation, we constructed a series of network models each incorporating different combinations of distinct neuronal cell types. For this purpose, cell types were differentiated by intrinsic properties (cellular time constants), circuit connectivity, neurotransmitter identity (excitatory or inhibitory), and their roles in circuit computation (feedforward or feedback). We first constructed a simple network with a single cell population of excitatory output neurons receiving feedforward excitation from a small number of afferent inputs (Figure 1A; see Methods). Given seven input units with binary activity (either 0 or 1), a combinatorial set of 2^7^ = 128 distinct input patterns was generated (Figure 1B). For each input pattern, the selected binary input units were activated continuously for a simulated duration of 350 ms, during which conductance, voltage, and activity dynamics were computed for all other cells. Thus, input patterns were distinguished only by the number and identity of active input units rather than by any differences in firing rate or temporal dynamics.

Mimicking the large expansion of neuronal units from cortex to the hippocampal dentate gyrus, we included a total of 128 output units, one for each distinct input pattern. In the ideal case, each input pattern would activate only one single output neuron (Figure 1C), as this representation would be maximally sparse (for each pattern, a minimum fraction of the population would have nonzero activity), selective (each unit would have nonzero activity for a minimum fraction of the presented patterns), and discriminable (output activity would have minimal overlap across different patterns). Note that we do not expect this ideal output representation to emerge without fine-tuning of the synaptic weights, which would require a learning process or network training procedure. Rather, here we sought to determine how closely the network output activities can approach this idealized target when initialized with random connection strengths, before learning. This will then establish a baseline for comparison to understand the impact of including additional neuronal cell types in the network.

First we sampled synaptic weights from a uniform distribution (Figure 1D) that ranged from zero to a maximum weight value obtained by an optimization procedure that aimed to maximize metrics of sparsity, selectivity, and discriminability of the output activity patterns. These metrics were based on quantifications used in previous studies of pattern separation and population coding (Willmore and Tolhurst, 2001; Berkes et al., 2009; Myers and Scharfman, 2009; Braganza et al., 2020) and are described in more detail in the Methods section. Neuronal units were implemented as single-compartment leaky integrators of synaptic currents generated by saturable conductance-based synapses. The activity of each output unit was either zero if the weighted sum of its inputs was below an activation threshold, or varied up to a saturating maximum value of one (see Methods). Since excitatory synaptic weights were all positive, as the number of active inputs grew across different patterns, the number of active output units also increased (Figures 1E,G). Compared to the input units, which were each active for exactly 50% of the patterns, the majority of the output units in this network with uniform synaptic weights exhibited nonzero activity for the majority of presented patterns (Figure 1I), which was far from the target ideal output.

We next considered a network with synaptic weights sampled instead from a log-normal distribution (Figure 1D). Experimental evidence indicates that many hippocampal and cortical neurons contain such skewed distributions of synaptic weights such that the average synaptic strength is weak, but a minority of synapses have strengths much greater than the average (Buzsáki and Mizuseki, 2014). This could enable neurons to exhibit a high degree of selectivity for a minority of stimuli (de Almeida et al., 2009; Grienberger et al., 2017; Rubin et al., 2017). Indeed, tuning the mean synaptic strength in this network resulted in output activities with fewer active units per pattern (Figures 1F,G), and with a higher proportion of output units responding to a minority of patterns compared to either the inputs, or the network with uniform weights (Figures 1F,I). For each of these network configurations, simulations were repeated for five instances of each network where synaptic weights were independently sampled from the same random distributions (see Methods). For each input pattern, we computed a sparsity metric as the complement of the fraction of active units (Figure 1H), and for each unit, we computed a selectivity metric as the complement of the fraction of active patterns (Figure 1J). As exceptions, patterns with zero active units were considered to have a sparsity value of zero rather than one, and units with zero active patterns were considered to have a selectivity value of zero instead of one (Supplementary Figure S1; see Methods). Comparing the distributions of sparsity values across patterns, and the distributions of selectivity values across units, the network with log-normal weights exhibited increased sparsity and selectivity relative to both the inputs and the network with uniform weights (Figures 1H,J).

**Figure 2:**
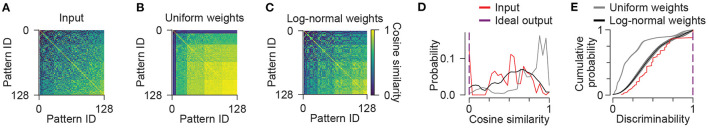
Pattern discrimination. **(A–C)** For each pair of input patterns, the similarity of the activities of each population is computed using the cosine similarity metric. Representational similarity matrices are shown for input units **(A)**, and output units in the network with uniform **(B)** or log-normal weights **(C)**. **(D)** Cosine similarity across all pairs of input patterns is shown as histograms for input units (red), and output units in the network with uniform (gray) or log-normal (black) synaptic weights. Ideal output similarity (purple) is shown for reference. Data shown in **(A–D)** are from single representative network instances of the network. **(E)** Across all pairs of patterns, discriminability is shown as cumulative probability distributions. Solid lines and shading indicate mean and standard deviation across five network instances (Uniform vs. Input: *p* < 0.001; Log-normal vs. Input: *p* = 0.0166; Log-normal vs. Uniform: *p* < 0.001). Ideal discriminability (purple) is shown for reference. Statistical comparisons reflect two-sample two-tailed Kolmogorov-Smirnov tests with *p*-values adjusted by Bonferroni correction for multiple comparisons.

In order to quantify how discriminable patterns of neuronal activity were from each other, we considered the activity of a population to be a vector with each element corresponding to one unit in the population. We computed the angle between each pair of activity vectors using a metric called cosine similarity (see Methods). The input patterns themselves ranged in similarity, with patterns consisting of only a single active unit being dissimilar from most other patterns, and with patterns consisting of a high number of active units being highly similar to many other patterns (Figures 2A,D). In contrast, the patterns of activity produced by the output population in the network with uniform weights were even more similar to each other, as the activity of most units correlated with the number, rather than the identity of active inputs (Figures 2B,D). The output activities of the network with log-normal weights were less similar across patterns compared to the network with uniform weights, and were comparable to the inputs themselves (Figures 2C,D). For each pair of patterns, we also computed a discriminability metric as the complement of cosine similarity, with an exception that patterns with zero active units were considered to have a discriminability of zero rather than one (Figure 2E, Supplementary Figure S1; see Methods). When averaged across network instances, the distribution of discriminability values was higher for the network with log-normal weights compared to the network with uniform weights, but both were reduced compared to the discriminability of the inputs (Figure 2E).

In summary, skewed initial distributions of excitatory synaptic weights help to promote and enhance the sparsity, selectivity, and discriminability of highly similar patterns of inputs. We next tested whether the addition of specific classes of inhibitory interneurons to the network can further improve these metrics of pattern separation.

### 2.2. Feedforward Inhibition

**Figure 3:**
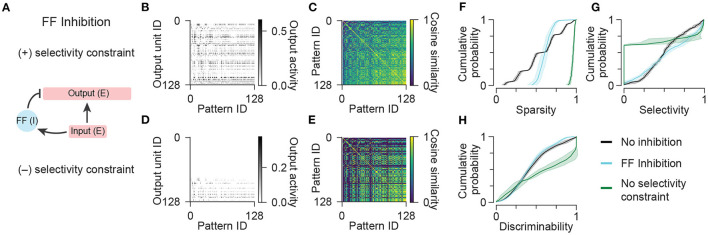
Network models with feedforward inhibition. **(A)** Diagram illustrating the connectivity of a simple model network containing populations of excitatory and feedforward inhibitory neurons. **(B)** Activity of output units in the network with feedforward inhibition in response to all input patterns. **(C)** Representational similarity matrix is shown for output units in the network with feedforward inhibition. **(D,E)** Same as **(B,C)** for a variant of the network with feedforward inhibition optimized without a constraint on the pattern selectivity of output units. Data shown in **(B–E)** are from single representative network instances of the network. **(F)** Population sparsity across all input patterns is shown as cumulative probability distributions (FF Inhibition vs. No inhibition: *p* < 0.001; No selectivity constraint vs. No inhibition: *p* < 0.001; FF Inhibition vs. No selectivity constraint: *p* < 0.001). **(G)** Pattern selectivity across all output units is shown as cumulative probability distributions (FF Inhibition vs. No inhibition: *p* < 0.001; No selectivity constraint vs. No inhibition: *p* < 0.001; FF Inhibition vs. No selectivity constraint: *p* < 0.001). **(H)** Output pattern discriminability is shown as cumulative probability distributions (FF Inhibition vs. No inhibition: *p* = 0.292; No selectivity constraint vs. No inhibition: *p* < 0.001; FF Inhibition vs. No selectivity constraint: *p* < 0.001). In **(F–H)**, solid lines and shading indicate mean and standard deviation across five network instances. Statistical comparisons reflect two-sample two-tailed Kolmogorov-Smirnov tests with *p*-values adjusted by Bonferroni correction for multiple comparisons.

In accordance with hippocampal and cortical circuits in which excitatory neurons far outnumber inhibitory neurons (Tremblay et al., 2016; Pelkey et al., 2017), we introduced a small population (7 units) of feedforward inhibitory neurons into the network (Figure 3A) to examine the impact of this circuit element on the generation of unique patterns of output given highly similar patterns of input (Figure 1A). We again sampled excitatory input weights onto output cells from a log-normal distribution (Figure 1D), and now sampled excitatory input weights onto feedforward interneurons and inhibitory weights from interneurons onto output cells from uniform distributions. This is consistent with experimental observations that hippocampal inhibitory neurons have reduced stimulus selectivity compared to excitatory neurons, and inhibitory conductances onto hippocampal excitatory neurons exhibit less heterogeneity than excitatory conductances (Grienberger et al., 2017). The mean weight of each projection between cell types was optimized to maximize sparsity, selectivity, and discriminability (see Methods). This resulted in an increased proportion of output activity patterns with high sparsity, and an increased proportion of output units with high selectivity compared to the network with no inhibitory elements (Figures 3B,F,G; “No inhibition” condition duplicated from “Log-normal weights” condition in Figure 1). These results are consistent with the above-mentioned “background subtraction” function of feedforward inhibition, which enables the total output activity to be less sensitive to the total input activity.

However, the addition of feedforward inhibitory interneurons did not result in any improvement in pattern discriminability (Figures 3C,H). This suggested that this network configuration resulted in many of the same units participating in representing the same patterns. We wondered if the relative strength of inhibition was increased, if the number of active units per pattern could be further decreased and pattern discriminability could be increased. However, we found that models with increased ratios of inhibition to excitation often completely suppressed the activity of some units such that they did not respond to any input patterns, and thus exhibited zero selectivity. To demonstrate this, we removed the selectively criterion during optimization entirely and analyzed the resulting patterns of output activity (Figure 3D). Indeed, this network configuration demonstrated the capability of feedforward inhibition to greatly increase sparsity (Figure 3F) and improve discriminability (Figures 3E,H), but it came at the extreme cost of decreasing the selectivity of individual output units (Figure 3G), the majority of which became silenced and did not participate in representing any of the patterns (Figure 3D). These results show that, under conditions of large variance in the number of active inputs, feedforward inhibition is able to normalize the number of active outputs, but is unable to improve overall pattern discriminability without decreasing the participation and selectivity of excitatory output neurons.

### 2.3. Feedback Inhibition

**Figure 4:**
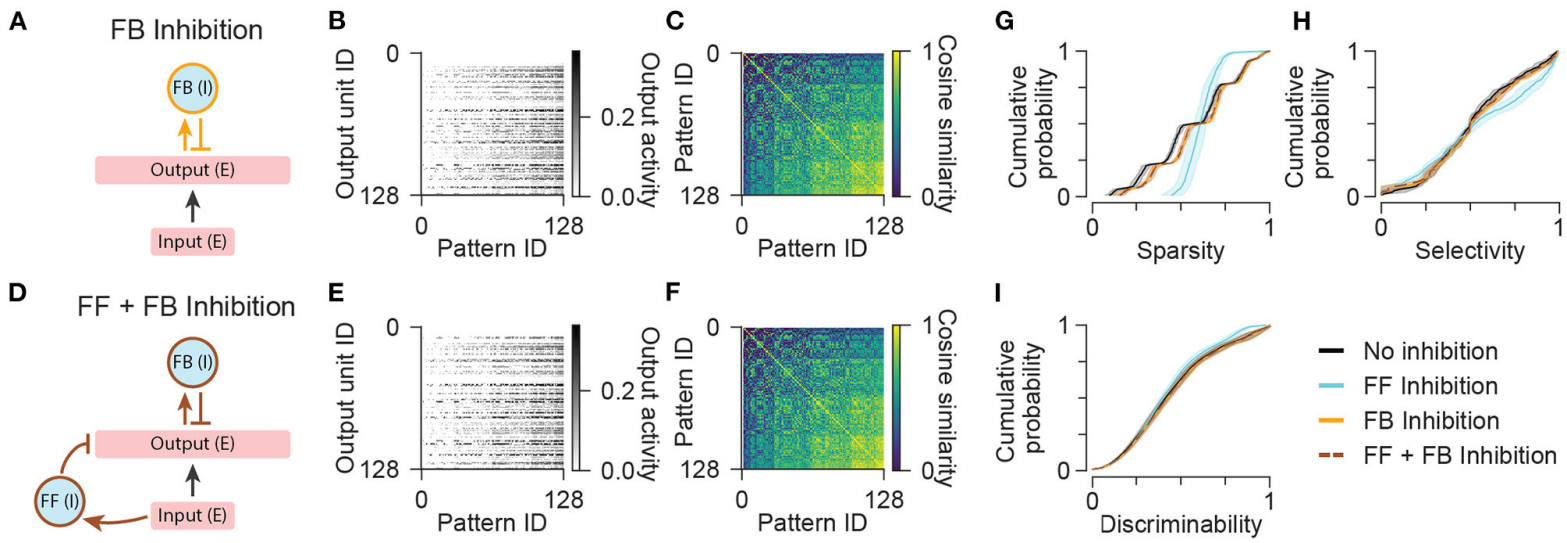
Network models with feedback inhibition. **(A)** Diagram illustrating the network configuration with only feedback inhibition. **(B)** Activity of output units in the network with only feedback inhibition in response to all input patterns. **(C)** Representational similarity matrix is shown for output units in the network with only feedback inhibition. **(D)** Diagram illustrating the network configuration with both feedforward and feedback inhibition. **(E,F)** Same as **(B,C)** for the network with both feedforward and feedback inhibition. Data shown in **(B,C,E,F)** are from single representative network instances of the network. **(G)** Population sparsity across all input patterns is shown as cumulative probability distributions (FF Inhibition vs. No inhibition: *p* < 0.001; FB Inhibition vs. No inhibition: *p* < 0.001; FF + FB Inhibition vs. No inhibition: *p* < 0.001; FB Inhibition vs. FF Inhibition: *p* < 0.001; FF + FB Inhibition vs. FF Inhibition: *p* < 0.001; FF + FB Inhibition vs. FB Inhibition: *p* = 1.00). **(H)** Pattern selectivity across all output units is shown as cumulative probability distributions (FF Inhibition vs. No inhibition: *p* < 0.001; FB Inhibition vs. No inhibition: *p* = 0.509; FF + FB Inhibition vs. No inhibition: *p* = 0.666; FB Inhibition vs. FF Inhibition: *p* < 0.001; FF + FB Inhibition vs. FF Inhibition: *p* < 0.001; FF + FB Inhibition vs. FB Inhibition: *p* = 1.00). **(I)** Output pattern discriminability is shown as cumulative probability distributions (FF Inhibition vs. No inhibition: *p* = 0.292; FB Inhibition vs. No inhibition: *p* = 1.00; FF + FB Inhibition vs. No inhibition: *p* = 1.00; FB Inhibition vs. FF Inhibition: *p* = 0.198; FF + FB Inhibition vs. FF Inhibition: *p* = 0.226; FF + FB Inhibition vs. FB Inhibition: *p* = 1.00). In **(G–I)**, solid lines and shading indicate mean and standard deviation across five network instances. Statistical comparisons reflect two-sample two-tailed Kolmogorov-Smirnov tests with *p*-values adjusted by Bonferroni correction for multiple comparisons.

The above results suggest that additional mechanisms besides feedforward inhibition may be required to ensure that different output units are activated by different patterns of inputs. Previously, it has been shown that feedback inhibition can support pattern separation by implementing a “winner-take-all” competition between output units. Within this framework, those output units that receive the most excitation recruit feedback inhibition, which then prevents the majority of other units from crossing threshold for activation. However, in previous models of feedback inhibition, the number of active output neurons typically scales with the amount of excitation from afferent inputs, so it is not clear whether this mechanism alone can support ultrasparse representations across a range of input activity levels (de Almeida et al., 2009; Rennó-Costa et al., 2019). Thus, we tested the effects of including a small population (7 units) of feedback inhibitory neurons, either alone or in combination with a separate population of feedforward inhibitory neurons (Figures 4A,D), on output sparsity, selectivity, and discriminability.

In the network with feedback inhibition alone, the average number of active units per pattern was slightly reduced compared to the network with no inhibition (Figures 4B,G), but total output activity was not prevented from increasing in proportion to the number of active inputs. This reflects a tension between maximizing sparsity at the highest input level while maintaining a minimum nonzero number of active units for the lowest input level. Without silencing the entire output population for any patterns or silencing any units across all patterns, feedback inhibition was unable to increase either the selectivity of output units or the discriminability of output patterns (Figures 4B,C,H,I). This limitation also constrained the performance of a network with both feedforward and feedback inhibitory populations such that the network exhibited lower degrees of sparsity and selectivity than the network with only feedforward inhibition (Figures 4D–H). Note that during optimization of networks with multiple cell populations, we imposed additional constraints on the activity of interneurons to ensure that the activity of each interneuron population was nonzero for the majority of input patterns (Methods). This prevented the network with both feedforward and feedback inhibitory interneurons from simply silencing the feedback interneurons to achieve the performance of the network with only feedforward inhibition. In summary, when simple networks are challenged to represent inputs across a wide range of activity levels with a consistently low number of active output units, canonical feedforward and feedback inhibitory neuronal populations have limited ability to improve pattern separation without silencing units or suppressing the activity of the entire output population when total input activity is low.

### 2.4. Dedicated Recurrent Excitatory Feedback Interneurons (Mossy Cells)

The above results suggest that standard feedforward and feedback inhibitory neuronal circuit motifs may be insufficient to support a maximally sparse stimulus representation. Feedforward inhibition that is too strong silences output units at the lowest input levels, and ultrasparse activity in an excitatory output population may be too low to provide enough excitation to drive feedback inhibitory cells beyond their activation threshold. Within the hippocampus, this problem may be unique to the dentate gyrus, as the fraction of output neurons that are active in a given spatial context in the CA3 and CA1 areas of the hippocampus are reported to be closer to 20–30%, compared to 2–5% for dentate granule cells (Hainmueller and Bartos, 2018). Interestingly, the dentate gyrus circuit includes an additional cell population not present in the other hippocampal regions, the mossy cells, which has features that could be beneficial for pattern separation (Myers and Scharfman, 2009). These cells are recurrently connected feedback excitatory interneurons, which could potentially increase the activity of granule cells *via* a direct excitatory connection (Ratzliff et al., 2004), or decrease their activity indirectly by activating local feedback inhibitory neurons (Scharfman, 1995; Bui et al., 2018). First, we tested whether this latter indirect feedback inhibitory function of mossy cells could improve pattern discrimination.

**Figure 5:**
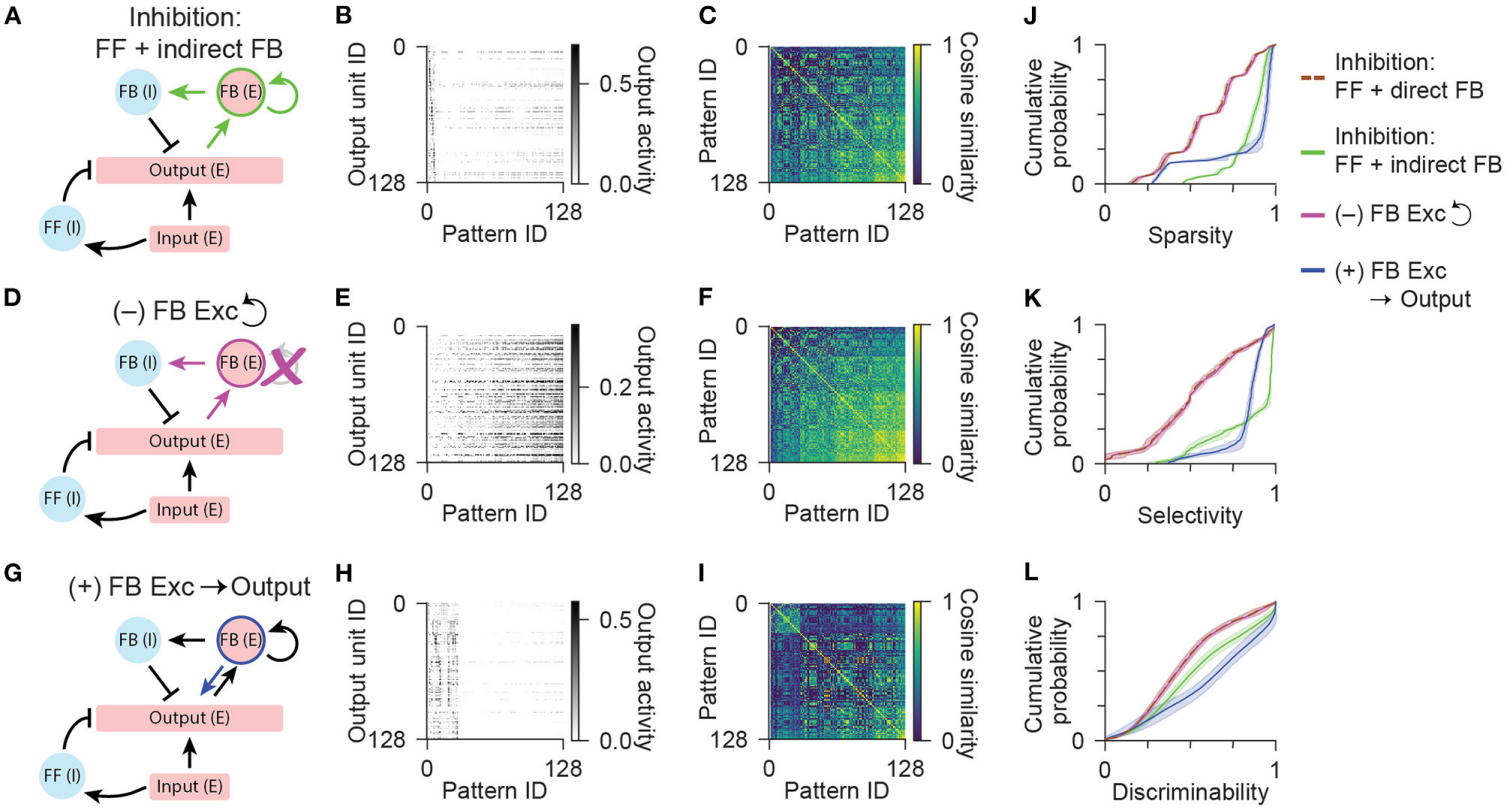
Network models with an excitatory feedback population. **(A)** Diagram illustrating the network configuration with indirect feedback inhibition, acting through an excitatory feedback interneuron population. **(B)** Activity of output units in the network with feedforward and indirect feedback inhibition in response to all input patterns. **(C)** Representational similarity matrix is shown for output units in the network with feedforward and indirect feedback inhibition. **(D)** Diagram illustrating a variant of the network configuration shown in **(A)** without recurrent connections between excitatory feedback interneurons. **(E,F)** Same as **(B,C)** for the network without recurrent connections between excitatory feedback interneurons. **(G)** Diagram illustrating a variant of the network configuration shown in **(A)** with an additional connection from feedback excitatory interneurons to the output population. **(H,I)** Same as **(B,C)** for the network with an additional connection from feedback excitatory interneurons to the output population. Data shown in **(B,C,E,F,H,I)** are from single representative network instances of the network. **(J)** Population sparsity across all input patterns is shown as cumulative probability distributions (FF + indirect FB vs. FF + direct FB: *p* < 0.001; No recurrent vs. FF + direct FB: *p* = 0.413; FB Excitation vs. FF + direct FB: *p* < 0.001; No recurrent vs. FF + indirect FB: *p* < 0.001; FB Excitation vs. FF + indirect FB: *p* < 0.001). **(K)** Pattern selectivity across all output units is shown as cumulative probability distributions (FF + indirect FB vs. FF + direct FB: *p* < 0.001; No recurrent vs. FF + direct FB: *p* = 0.996; FB Excitation vs. FF + direct FB: *p* < 0.001; No recurrent vs. FF + indirect FB: *p* < 0.001; FB Excitation vs. FF + indirect FB: *p* < 0.001). **(L)** Output pattern discriminability is shown as cumulative probability distributions (FF + indirect FB vs. FF + direct FB: *p* < 0.001; No recurrent vs. FF + direct FB: *p* = 1.00; FB Excitation vs. FF + direct FB: *p* < 0.001; No recurrent vs. FF + indirect FB: *p* < 0.001; FB Excitation vs. FF + indirect FB: *p* < 0.001). In **(J–L)**, solid lines and shading indicate mean and standard deviation across five network instances. Statistical comparisons reflect two-sample two-tailed Kolmogorov-Smirnov tests with *p*-values adjusted by Bonferroni correction for multiple comparisons.

While the previous network model with both feedforward and feedback inhibition contained a direct recurrent connection from excitatory output neurons to feedback inhibitory cells, here we removed that direct connection and replaced it with an indirect inhibitory pathway – output neurons provided input to a population (7) of mossy cell-like excitatory feedback interneurons, which then excited the feedback inhibitory cells (Figure 5A). Importantly, feedback excitatory cells were recurrently connected to each other, enabling small amounts of excitation from the output neurons to be amplified by this positive feedback loop. This network configuration exhibited marked improvements in all three metrics of pattern separation – sparsity, selectivity, and discriminability (Figures 5B,C,J,K,L). To test the hypothesis that this improvement depended on self-amplification by the recurrent connections between excitatory interneurons, we also tested a network configuration with this positive feedback connection removed (Figure 5D). Indeed, in the absence of this amplification mechanism, all metrics of pattern separation were decreased (Figures 5E,F,J,K,L). In fact, performance of this network was comparable to the network without excitatory feedback interneurons (Figures 5J–L; “FF + direct FB Inhibition” condition duplicated from “FF + FB Inhibition” condition in Figure 4). This indicated that simply adding an additional filter between excitatory output and inhibitory feedback is insufficient to reduce the fraction of active output neurons. Rather, the feedback excitatory interneurons helped by separating the dual roles of reporting sparse output to downstream areas, and providing dense excitation to local inhibitory neurons. Thus, ultrasparse activity of output neurons could be maintained by “offloading” the role of recruiting feedback inhibition to a dedicated excitatory interneuron population whose activity is not required to be sparse (Danielson et al., 2017; GoodSmith et al., 2017; Senzai and Buzsáki, 2017).

Finally, we sought to test if this function of excitatory interneurons could be reconciled with the additional role of dentate mossy cells in directly exciting the output granule neurons. By providing an additional source of dense excitation to the output cells that is less selective across patterns, this circuit element could potentially counteract the benefits of the indirect feedback inhibitory motif and actually reduce output sparsity and pattern separation. Importantly, we found that a network that incorporated all known output connections of dentate mossy cells, including the direct feedback excitatory connection to the output population (Figure 5G), was able to maintain a high degree of sparsity and selectivity (Figures 5H,J,K), and actually resulted in improved pattern discriminability relative to the network without this additional excitatory feedback connection (Figures 5I,L). These results indicate that the incorporation of a specialized excitatory feedback interneuron in the dentate gyrus supports the pattern separation function of the hippocampus by enabling overlapping patterns of input to activate maximally sparse and minimally overlapping patterns of output across a broad range of input activity levels.

## 3. Discussion

In this study we simulated and analyzed a series of simple neuronal network models incorporating different combinations of inhibitory and excitatory interneuron populations based on the neural circuit architecture of the rodent hippocampal dentate gyrus. We challenged these networks with a set of highly overlapping patterns of afferent input that spanned a wide range of total activity levels, and compared their abilities to produce unique patterns of output in response to each presented pattern. We found that standard feedforward and feedback inhibitory circuit motifs were insufficient to enable the excitatory output population in these networks to represent each stimulus with a minimal but nonzero number of active output units with minimal overlap across patterns. Interestingly, we found that incorporating into the network a dedicated recurrent excitatory interneuron modeled after the mossy cells of the dentate gyrus resulted in output patterns that were highly sparse and discriminable from each other. These specialized excitatory feedback interneurons received a copy of the sparse output of the circuit, increased their own activity *via* recurrent excitatory connections with each other, and then provided dense excitation to feedback inhibitory interneurons that in turn enforced a low fraction of active output neurons.

Our modeling results demonstrated that pattern separation was robust to inclusion of the direct excitatory feedback connection from mossy cells to granule cells, but that it was not required (but see Myers and Scharfman, 2009). While here we aimed to identify architectural priors that may enable biological neural circuits to initially perform pattern separation on sensory stimuli prior to learning, in future work it will be important to determine how the presence of excitatory feedback from mossy cells influences experience-dependent synaptic plasticity and pattern storage, which is expected to fine-tune synaptic strengths to improve output pattern discriminability even further. Interestingly, recent work showed that silencing of mossy cells during memory encoding degraded future recall (Bui et al., 2018). However, the activity of mossy cells was not required to recall a spatial memory that had previously been successfully encoded. This suggests that another function of mossy cells may be to promote, or “gate” synaptic plasticity in dentate granule cells, but that once the appropriate modifications in synaptic strength have been made to the cortical inputs to the granule cells, the mossy cells are not required for the appropriate sparse pattern to be recalled. Supporting this possibility, mossy cells preferentially synapse onto the proximal portion of granule cell dendrites (Buckmaster et al., 1996), making them well positioned to influence dendritic events. In particular, dendritic depolarization by mossy cell input could promote the generation of dendritic spikes, which have been shown to drive synaptic plasticity in granule cells (Kim et al., 2018). Compartmentalization and nonlinear integration of synaptic input in granule cell dendrites may also directly contribute to representational sparsity (Chavlis et al., 2017). Finally, in this study we did not explore the roles of temporal dynamics such as synaptic adaptation and population oscillations in pattern separation. Both synapses from granule cells onto mossy cells and from mossy cells onto granule cells exhibit low basal release probabilities that facilitate during bouts of high firing rate (Lysetskiy et al., 2005; Hashimotodani et al., 2017), and the firing rates of both granule cells and mossy cells have been shown to be modulated and entrained by hippocampal population oscillations in the theta (~4–10 Hz) and gamma (~30–80 Hz) frequency bands (Senzai and Buzsáki, 2017). These features may enable the dentate gyrus to additionally discriminate inputs at distinct frequencies (Braganza et al., 2020), meriting further investigation.

Overall, the simulation results presented here provide insight into how biological diversity of neuronal cell types expand the computational capabilities of neuronal circuits in the mammalian brain. In the case of the dentate gyrus, the pattern separation function of the circuit requires extremely sparse population activity, which limits the efficacy of standard winner-take-all competition enforced by the direct recruitment of feedback inhibition. This problem appears to have been resolved by inclusion of an additional neuronal cell type, the mossy cells. While analogous dedicated excitatory interneurons have not been found in other hippocampal or cortical circuit layers, other neuronal circuits do exhibit sparse sensory representations and feature prominent recurrent excitatory connections (Douglas et al., 1995; Olshausen and Field, 2004). It is possible that in other neuronal circuits, while most excitatory neurons do project outside the local circuit, local recurrent excitation that is appropriately balanced by strong local inhibition could perform a similar function as mossy cells to increase the discriminability of sensory stimuli (Rubin et al., 2017; Sadeh and Clopath, 2021).

## 4. Methods

### 4.1. Network Model Simulation

Computational models of neuronal circuits with a variety of cell populations based on the hippocampal dentate gyrus were implemented and simulated using custom code written in python 3.8. Input to each network model variant was provided by a population of 7 input units that could take on binary (0 or 1) activity values. All network models included a population of 128 output neurons, and some models included additional neuronal cell populations of 7 units from the following categories: feedforward inhibitory interneuron, feedback inhibitory interneuron, and feedback excitatory neuron (Table 1). All neuronal cell models were implemented as single-compartment leaky integrators with membrane voltage dynamics that evolved over time as follows:

(1) $$\begin{array}{l} \tau_{cell}\frac{dV}{dt}=-V+IR \end{array}$$

where τ_*cell*_ is the membrane time constant, *R* is the neuron's input resistance, and *I* is the total synaptic current received by each cell. For simplicity, all cells had a resting membrane voltage of 0 mV. Synaptic currents were generated through saturable conductance-based synapses described as:

(2) $$\begin{array}{l} I_{i}=\overset{n}{\underset{j=1}{\sum }}w_{ij}g_{ij}\left(E-V_{i}\right) \end{array}$$

where *g*_*ij*_ represents the normalized synaptic conductance from neuron *j* to neuron *i*, the synaptic weight *w*_*ij*_ is a synapse-specific scaling factor that determines the relative strength of each input, and *V*_*i*_ is the membrane potential of neuron *i*. The reversal potential *E* was set to +60 mV for excitatory synapses, and –10 mV for inhibitory synapses. When activity *a*_*j*_ in a presynaptic neuron *j* was nonzero, the synaptic conductance *g*_*ij*_ in neuron *i* increased with kinetics determined by exponential rise time constant τ_*rise*_ and saturated at the value of *a*_*j*_. Once activated, synaptic conductances decreased with kinetics determined by exponential decay time constant τ_*decay*_:

(3) $$\begin{array}{l} \frac{dg_{ij}}{dt}=\frac{-g_{ij}}{\tau_{decay}}+\frac{max\left(a_{j}-g_{ij},0\right)}{\tau_{rise}} \end{array}$$

Neuronal output activities were determined by a piecewise linear function of membrane voltage, such that output was zero for voltages less than an activation threshold of +10 mV, and increased linearly to a value of one at the reversal potential of excitatory synapses (+60 mV).

**Table 1:** Cell parameters used across all models.

**Parameter**	**Input**	**Output**	**FF Inh**	**FB Inh**	**FB Exc**
No. of units	7	128	7	7	7
τ_*cell*_	N/A	0.05	0.02	0.02	0.05
Synapse τ_*rise*_	0.001	0.001	0.001	0.001	0.001
Synapse τ_*decay*_	0.01	0.01	0.02	0.02	0.01
Synapse *E*_*rev*_	+60	+60	–10	–10	+60

*Synapse parameters apply to all connections from the specified population onto all other connected populations*.

Patterns of input were drawn from the combinatorial set of 128 permutations of the activities of the 7 binary input units (Figure 1A). One pattern of input activities was presented at a time for a simulation duration of 350 ms. The synaptic conductances, synaptic currents, intracellular voltages, and output activities of all neurons in the network model comprised a large system of coupled differential equations, which were solved by numerical approximation using an initial value problem solver implemented in the python package SciPy 1.5.2. Following an initial onset transient, the activities of the neurons in the network relaxed toward an equilibrium (Supplementary Figure S4). All analyses were performed using neuronal activity values for each unit that were averaged across time during the final 200 ms of each simulation.

### 4.2. Analysis of Pattern Separation

In previous studies of neuronal stimulus representations, a variety of metrics have been used to quantify pattern separation. Here we adopted three simple and easily interpretable metrics: population sparsity, unit selectivity, and pairwise pattern discriminability (Supplementary Figure S1), which were defined as follows. For each input pattern *k*, *sparsity*_*k*_ was defined as:

(4) $$\begin{array}{l} {sparsity}_{k}=\{\begin{array}{ll} 1-F_{units,k} & F_{units,k}>0\\ 0 & F_{units,k}=0 \end{array} \end{array}$$

where *F*_*units,k*_ is the fraction of units that had nonzero activity in response to the input pattern *k*. For each unit *i*, *selectivity*_*i*_ was defined as:

(5) $$\begin{array}{l} {selectivity}_{i}=\{\begin{array}{ll} 1-F_{patterns,i} & F_{patterns,i}>0\\ 0 & F_{patterns,i}=0 \end{array} \end{array}$$

where *F*_*patterns,i*_ is the fraction of patterns that the input *i* responded to with nonzero activity. For each pair of patterns *k* and *l*, *discriminability*_*k,l*_ was defined as:

(6) $$\begin{array}{l} {discriminability}_{k,l}=\{\begin{array}{ll} 1-C_{k,l} & F_{units,k}>0\&F_{units,l}>0\\ 0 & otherwise \end{array} \end{array}$$

where *C*_*k,l*_ is the cosine similarity between the two vectors of population activity generated in response to patterns *k* and *l*.

### 4.3. Optimization

For each pair of cell populations with synaptic connections, synaptic weights were sampled randomly from either a uniform distribution for inhibitory synapses and excitatory synapses onto inhibitory neurons, or a log-normal distribution for excitatory synapses onto excitatory neurons. For simplicity during model optimization, weight distributions were parameterized by their mean weight, with uniform distributions ranging from zero to twice the mean weight. Log-normal distributions were initially generated as the natural log of a random normal variable with zero mean and unit standard deviation, and then all sampled values were rescaled such that the mean of the sampled values was equal to the desired value. During optimization, the mean weight values for each projection were varied within bounds from 0.01 to 1 (Table 2).

**Table 2:** Model weights.

**Parameter (mean weight)**	**Bounds**	**No Inh (uniform)**	**No Inh (log-normal)**	**FF Inh**	**FF Inh (no selectivity constraint)**	
Input → Output	0.01–1	0.1135	0.0681	0.1905	0.1026	
Input → FF Inh	0.01–1			0.1336	0.2319	
FF Inh → Output	0.01–1			0.9996	0.7684	
**Parameter** **(mean weight)**	**Bounds**	**FB Inh**	**FF +** **direct** **FB Inh**	**FF +** **indirect** **FB Inh**	**(-) FB Exc** **→ FB Exc**	**(+) FB Exc** **→ Output**
Input → Output	0.01–1	0.0702	0.0775	0.3891	0.0763	0.2090
Input → FF Inh	0.01–1		0.9183	0.1640	0.9754	0.4825
FF Inh → Output	0.01–1		0.0125	0.3011	0.0109	0.0110
Output → FB Inh	0.01–1	0.9963	0.9920			
FB Inh → Output	0.01–1	0.0100	0.0100	0.9994	0.0111	0.9983
Output → FB Exc	0.01–1			0.0167	0.9216	0.0158
FB Exc → FB Exc	0.01–1			0.7891		0.9333
FB Exc → FB Inh	0.01–1			0.8583	0.9366	0.7726
FB Exc → Output	0.01–1					0.0423

Optimization was performed using an iterative population-based multi-objective algorithm based on simulated annealing (Wales and Scheraga, 1999). For each model tested with different mean weight parameters, objective costs were computed based on the above-described metrics of population sparsity, unit selectivity, and pattern discriminability. These objective error values were expressed as a sum of squared residuals after comparison to target values for maximum sparsity, selectivity, and discriminability. Each model was evaluated by presenting all 128 input patterns to each of 5 independent instances of the network, where in each instance synaptic weights were independently sampled from the same random weight distribution. Objective errors were then averaged across the 5 network instances. During each of 50 iterations, a population of 600 models with distinct parameters was simulated and evaluated. Within each iteration, the performance of models within a population were compared to each other and ranked with a non-dominated sorting procedure (Deb, 2011). Then, a new population of models was generated by randomly varying the parameter values of the most highly ranked models from the previous iteration. Models were not selected for further search if they did not meet the following additional inclusion criterion: for the output population, 90% of units must be active for at least one pattern, and 90% of patterns must have at least one active unit; for all interneuron populations, 80% of units must be active for at least one pattern, and 60% of patterns must have at least one active unit (see Supplementary Figures S2, S3). The final optimized parameter values for each tested model configuration are presented in Table 2.

## Data Availability Statement

The datasets presented in this study can be found in online repositories. The names of the repository/repositories and accession number(s) can be found below: https://github.com/Milstein-Lab/dentate_circuit_model.

## Author Contributions

AG contributed to conceptualization, methodology, software, validation, writing, and visualization. AS, KH, and RO contributed to methodology, software, validation, writing, and visualization. AM contributed to conceptualization, methodology, software, validation, writing, visualization, supervision, project administration, and funding acquisition. All authors contributed to the article and approved the submitted version.

## Funding

Undergraduate research at Rutgers University was generously supported by the following programs: Center for Advanced Technology and Medicine Summer Undergraduate Research Experience, Aresty Summer Science Program, and Department of Neuroscience and Cell Biology Neuroscience Summer Undergraduate Research Program. This work was funded by NIMH grant R01MH121979.

## Conflict of Interest

The authors declare that the research was conducted in the absence of any commercial or financial relationships that could be construed as a potential conflict of interest.

## Publisher's Note

All claims expressed in this article are solely those of the authors and do not necessarily represent those of their affiliated organizations, or those of the publisher, the editors and the reviewers. Any product that may be evaluated in this article, or claim that may be made by its manufacturer, is not guaranteed or endorsed by the publisher.
