# Supplementary material for: Recurrent Excitatory Feedback From Mossy Cells Enhances Sparsity and Pattern Separation in the Dentate Gyrus via Indirect Feedback Inhibition
Source: Front Comput Neurosci. 2022 Feb 10;16:826278. doi: 10.3389/fncom.2022.826278 (PMC8866186; doi:10.3389/fncom.2022.826278)
Supplement: Supplementary file 1 [file Data_Sheet_1.PDF]

# Supplementary Material

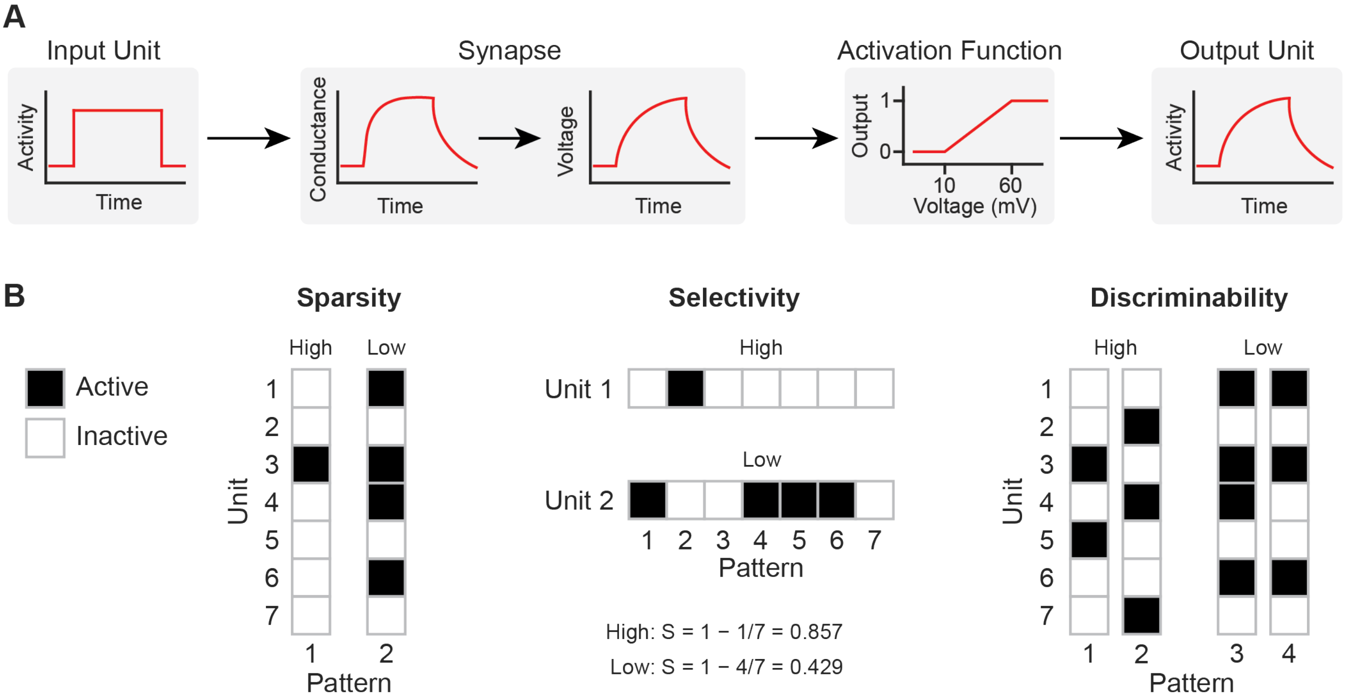


**Figure S1. Network activity dynamics and metrics of pattern separation.** **(A)** Illustration of computational processing pipeline during feedforward dynamics. A constant binary pattern from the input units is temporally filtered through both the synaptic and cellular time constants to produce a time-varying voltage in the postsynaptic units. This voltage is then passed through a piecewise linear activation function to determine a unit's output, which is passed to downstream neurons through a similar process. **(B)** Examples illustrating the three metrics of pattern separation (sparsity, selectivity, and discriminability) with example activity patterns. *Left:* Population activity patterns with high and low sparsity. *Middle:* Activity across all patterns for individual units with high and low selectivity. *Right:* Pairs of population activity patterns with high and low discriminability.

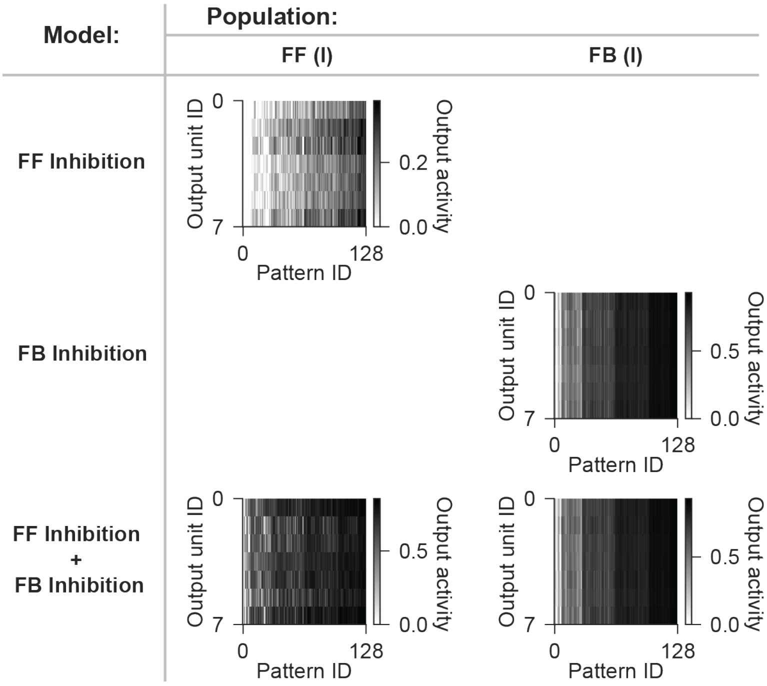


**Figure S2. Activities of interneuron populations in models with inhibitory interneurons.** FF (left) and FB (right) inhibitory population activities across all patterns in the models with only FF inhibition (top row), FB inhibition (middle row), or both (bottom row).

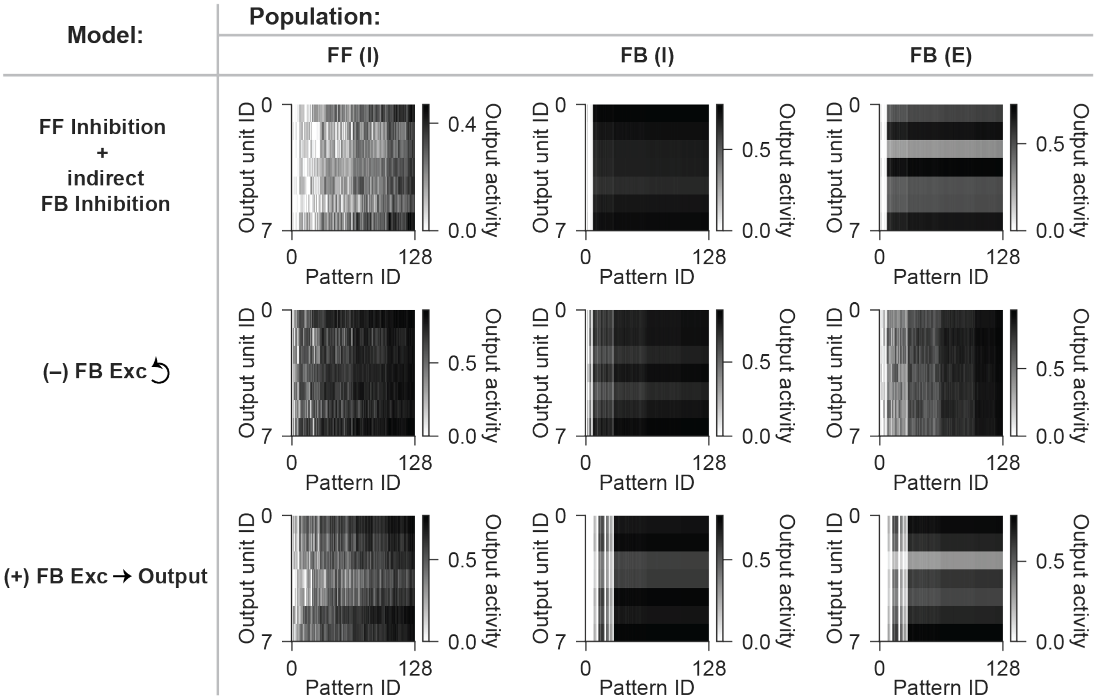


**Figure S3. Activities of interneuron populations in models with excitatory interneurons.** Activity of FF (left) and FB (middle) inhibitory populations and FB excitatory (right) populations across all patterns in the models with indirect feedback inhibition (top row), without recurrent excitatory connections (middle row), and with direct FB excitation of the output units (bottom row).

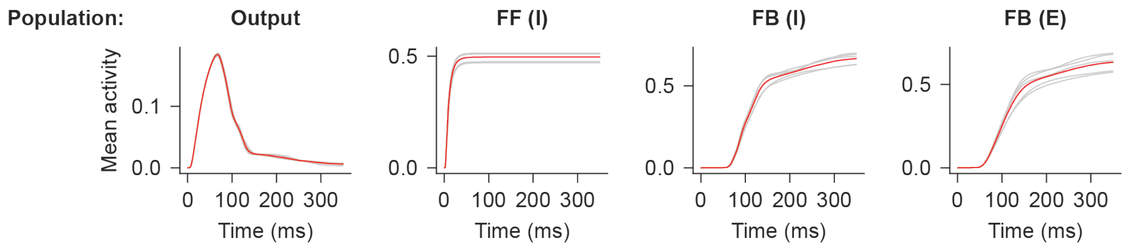


**Figure S4. Temporal dynamics.** Mean activity dynamics of each population over the course of the 350 ms simulation. Each time point represents an average across both patterns and units for each population. Each of five network instances are shown in gray, and averages across network instances are shown in red.
